# Contribution of the Type VI Secretion System Encoded in SPI-19 to Chicken Colonization by *Salmonella enterica* Serotypes Gallinarum and Enteritidis

**DOI:** 10.1371/journal.pone.0011724

**Published:** 2010-07-22

**Authors:** Carlos J. Blondel, Hee-Jeong Yang, Benjamín Castro, Sebastián Chiang, Cecilia S. Toro, Mercedes Zaldívar, Inés Contreras, Helene L. Andrews-Polymenis, Carlos A. Santiviago

**Affiliations:** 1 Departamento de Bioquímica y Biología Molecular, Facultad de Ciencias Químicas y Farmacéuticas, Universidad de Chile, Santiago, Chile; 2 Department of Microbial and Molecular Pathogenesis, College of Medicine, Texas A&M University System Health Science Center, College Station, Texas, United States of America; 3 Programa de Microbiología y Micología, Instituto de Ciencias Biomédicas, Facultad de Medicina, Universidad de Chile, Santiago, Chile; National Institutes of Health, United States of America

## Abstract

*Salmonella* Gallinarum is a pathogen with a host range specific to poultry, while *Salmonella* Enteritidis is a broad host range pathogen that colonizes poultry sub-clinically but is a leading cause of gastrointestinal salmonellosis in humans and many other species. Despite recent advances in our understanding of the complex interplay between *Salmonella* and their hosts, the molecular basis of host range restriction and unique pathobiology of Gallinarum remain largely unknown. Type VI Secretion System (T6SS) represents a new paradigm of protein secretion that is critical for the pathogenesis of many Gram-negative bacteria. We recently identified a putative T6SS in the *Salmonella* Pathogenicity Island 19 (SPI-19) of Gallinarum. In Enteritidis, SPI-19 is a degenerate element that has lost most of the T6SS functions encoded in the island. In this work, we studied the contribution of SPI-19 to the colonization of *Salmonella* Gallinarum strain 287/91 in chickens. Non-polar deletion mutants of SPI-19 and the *clpV* gene, an essential T6SS component, colonized the ileum, ceca, liver and spleen of White Leghorn chicks poorly compared to the wild-type strain after oral inoculation. Return of SPI-19 to the ΔSPI-19 mutant, using VEX-Capture, complemented this colonization defect. In contrast, transfer of SPI-19 from Gallinarum to Enteritidis resulted in transient increase in the colonization of the ileum, liver and spleen at day 1 post-infection, but at days 3 and 5 post-infection a strong colonization defect of the gut and internal organs of the experimentally infected chickens was observed. Our data indicate that SPI-19 and the T6SS encoded in this region contribute to the colonization of the gastrointestinal tract and internal organs of chickens by *Salmonella* Gallinarum and suggest that degradation of SPI-19 T6SS in *Salmonella* Enteritidis conferred an advantage in colonization of the avian host.

## Introduction

*Salmonella enterica* contains over 2,500 diverse serotypes that have different host ranges, and cause diseases with severity ranging from subclinical colonization to serious systemic disease. Some highly prevalent serotypes including Typhimurium and Enteritidis, two isolates that cause the majority of human gastrointestinal salmonellosis, have a broad host range and are able to infect humans, livestock, and poultry colonizing the intestinal tract. Other serotypes have host range that is restricted to a single species.

*S.* Gallinarum has a host range restricted to birds and causes a severe systemic disease called fowl typhoid. Fowl typhoid is a disease of poultry characterized by high morbidity and mortality, and it causes major economic losses in poultry production in some parts of the world [1]. *S.* Gallinarum has been eradicated from developed countries such as the United States, Australia and many Western European countries as a result of improved surveillance and slaughter practices, but it is still a major concern in developing countries [1].

*S.* Enteritidis on the other hand, infects a broad range of hosts including humans, mice and avian species. In contrast to *S.* Gallinarum, *S.* Enteritidis generates a subclinical infection in poultry, and infected hens can become chronic carriers and produce eggs contaminated with *Salmonella* during oviposition [2], [3]. Human consumption of contaminated poultry or egg products results in an acute self-limiting gastroenteritis. *S.* Enteritidis accounts for ∼61% of the estimated 1.5 million human salmonellosis cases reported between 1995 and 2008 (WHO Global Foodborne Infections Network Country Databank http://www.who.int/salmsurv).

Recent work has identified potential virulence determinants that may be responsible for differences in pathogenesis and host-adaptation of serotypes Gallinarum and Enteritidis in poultry [4], [5]. Comparative analysis of the genomes of *S.* Gallinarum and *S.* Enteritidis suggests that Gallinarum is a direct descendant of Enteritidis that has become host-adapted to birds [5]. Consistent with this finding, the Gallinarum genome contains a large number of pseudogenes, suggesting that it has undergone genome degradation during adaptation to a single host as seen in other highly host-adapted *Salmonellae* [5], [6], [7].

In addition to loss of gene function, the genome of *S.* Gallinarum has several genomic islands that appear absent or degenerate in *S.* Enteritidis [5]. Variations in gene content at these regions, some of which encode putative virulence determinants, may also contribute to the differential pathobiology and host-restriction of these serotypes. Gallinarum may rely on a set of virulence determinants to infect avian hosts that are different from the classical repertoire of virulence factors commonly associated with most *Salmonella enterica* serotypes [8], [9], [10].

Type VI Secretion System (T6SS) represents an emerging paradigm for protein secretion in Gram-negative bacteria. T6SSs have been described in many bacterial pathogens and have been linked to a wide variety of functions ranging from interbacterial relationships and biofilm formation, to cytotoxicity and survival in phagocytic cells [11], [12], [13]. Most T6SS components correspond to structural elements of the secretion apparatus, including DotU and IcmF orthologs believed to stabilize the multiprotein complex in the membrane [14], [15] and a ClpV homolog, a member of the AAA+ family of ATPases hypothesized to energize the system for protein secretion [16], [17], [18]. Functionality of T6SS is often defined by the detection of the structural/secreted components Hcp and VgrG in culture supernatants [17], [19], [20]. These proteins resemble structural components of the T4 bacteriophage tail tube and tail spike, and are believed to be extracellular components of the secretion machinery [21].

**Figure 1:**
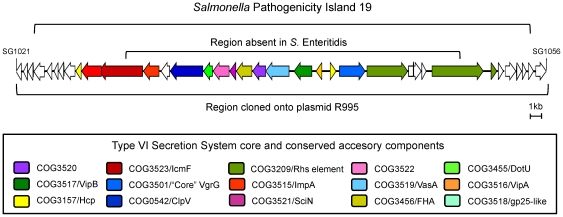
Schematic representation of *Salmonella* Pathogenicity Island 19 in *S.* Gallinarum strain 287/91. The SPI-19 (ORFs SG1021 to SG1056), the T6SS gene cluster, the region that is absent in serotype Enteritidis and the region cloned onto plasmid R995 by the VEX-Capture technique are shown in different brackets. ORFs are shown in arrows and those which encode conserved T6SS components are shown with different colors.

Four phylogenetically distinct T6SS loci are differentially distributed among *Salmonella enterica* serotypes [22]. One such system is encoded in the *Salmonella* Pathogenicity Island 19 (SPI-19) present in serotypes Dublin, Weltevreden, Gallinarum and Enteritidis. Interestingly, while *S.* Gallinarum appears to encode a complete T6SS in SPI-19 (**Figure 1**), *S.* Enteritidis SPI-19 bears a degenerate genetic element lacking most of the T6SS related components [22]. Thus, it seems unlikely that the SPI-19-encoded T6SS in Enteritidis is functional.

In this work, we studied the contribution of the T6SS encoded in SPI-19 to the virulence of *S.* Gallinarum strain 287/91, and its ability to colonize chicks. We demonstrate that SPI-19, and the T6SS encoded there, contribute to the colonization of the gastrointestinal tract and the internal organs of chickens infected with *S.* Gallinarum. In addition, we show that transfer of a complete SPI-19 to *S.* Enteritidis has an overall negative effect on the colonization ability of this serotype in chicks.

## Results

### *S.* Gallinarum strain 287/91 efficiently colonizes the gut and internal organs of experimentally infected chickens

**Figure 2:**
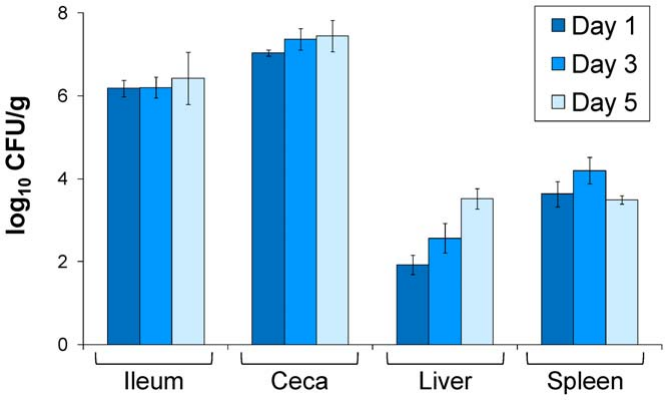
Distribution of *S.* Gallinarum strain 287/91 in the gastrointestinal tract and internal organs of orally infected chickens. White Leghorn chicks were orally infected with ∼10^9^ CFU of the wild-type *S*. Gallinarum 287/91 strain. After 1, 3 and 5 days post-infection the chicks were humanely euthanized, the ileum, ceca, liver and spleen were aseptically removed, tissues were homogenized and viable bacterial counts were determined. Data are mean values of log_10_ CFU/g of tissue, from four animals at each time point.

Strain 287/91 is currently the only sequenced strain of serotype Gallinarum [5]. Although this isolate is reported to be highly virulent in susceptible chickens [5], there are no published reports regarding the systemic colonization dynamics of this strain in experimentally infected chickens. We infected White Leghorn chicks at four days of age with ∼1.39×10^9^ CFU of the wild-type 287/91 strain by the oral route. The degree of colonization of the ileum, ceca, liver and spleen of infected chickens was determined at 1, 3 and 5 days post-infection (**Figure 2**). Strain 287/91 efficiently colonized the gut and internal organs of infected chicks from day 1 post-infection, gradually increasing during the remaining time points. At 5 days post infection the chicks were heavily colonized by *S.* Gallinarum but there were no noticeable differences in gross pathology with the uninfected control group and infected chicks did not show appreciable clinical signs of fowl typhoid. Nevertheless, since strain 287/91 efficiently colonized both the gut and internal organs of infected chickens, we used it to determine the contribution of SPI-19 to the colonization of chickens by *S.* Gallinarum.

### The T6SS encoded in SPI-19 contributes to the colonization of chickens by *S.* Gallinarum strain 287/91

**Figure 3:**
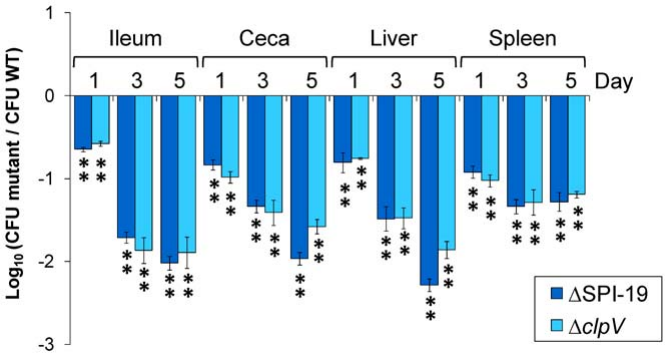
Competitive index experiments with ΔSPI-19 and Δ*clpV* mutants versus the wild-type *S.* Gallinarum strain 287/91. Four day old White Leghorn chicks were orally infected with ∼10^9 ^CFU of an approximately equal mixture of the mutant strains and the wild-type Gallinarum 287/91 strain. After 1, 3 and 5 days post-infection the chicks were humanely euthanized, the ileum, ceca, liver and spleen were recovered. Tissues were homogenized and viable counts were determined after serial dilution of the homogenates and plating on appropriate media. Error bars represent standard error. Statistical significance was determined using a two-tailed Student's *t* test. Asterisks indicate that normalized output ratios were significantly statistically different from the equivalent ratio in the inoculum. ** *P* value of <0.001, * *P* value of <0.05.

To evaluate the role played by SPI-19 in the colonization of chickens by *S.* Gallinarum, a ΔSPI-19 derivative of strain 287/91 was constructed and tested in a competitive index experiment. White Leghorn chicks were orally infected with ∼10^9^ CFU of a 1∶1 mixture of the mutant and the parental wild-type strain at four days post-hatch. Birds were euthanized at 1, 3, and 5 days post infection, bacteria were recovered from the ileum, ceca, liver and spleen of infected birds and the ability of the mutant to compete for organ colonization against the wild-type parental isolate was evaluated (**Figure 3**). The ΔSPI-19 mutant had a deficit in colonization of the intestine immediately after infection that became more severe throughout the duration of the experiment. ΔSPI-19 mutants also had reduced colonization of systemic organs, including the liver and spleen, of chicks at all time points post-infection.

We hypothesized that the colonization defect we observed for the ΔSPI-19 mutant strain could be attributable to the lack of T6SS function. To test this hypothesis, a non-polar Δ*clpV* derivative of *S.* Gallinarum 287/91 was generated using Lambda-Red recombination [23]. ClpV is a key component of the T6SS and has been suggested to provide the energy needed for type VI secretion in other systems [16], [17]. Consistent with this view, Δ*clpV* mutants are unable to secrete and translocate the hallmark structural/secreted T6SS components Hcp and VgrG in a wide range of bacteria [16], [24]. We determined that Δ*clpV* mutants have a strong colonization defect in the chick intestine and systemic organs (**Figure 3**) in competitive infection assays against the parental wild-type isolate. The colonization defect of the Δ*clpV* mutant was nearly identical to that of the ΔSPI-19 mutant. These results strongly suggest that a functional T6SS encoded in SPI-19 in *S*. Gallinarum strain 287/91 is required for efficient colonization of the gut and internal organs of chicks.

### Transfer of SPI-19 restores the ability of the *S.* Gallinarum ΔSPI-19 mutant to colonize chickens

**Figure 4:**
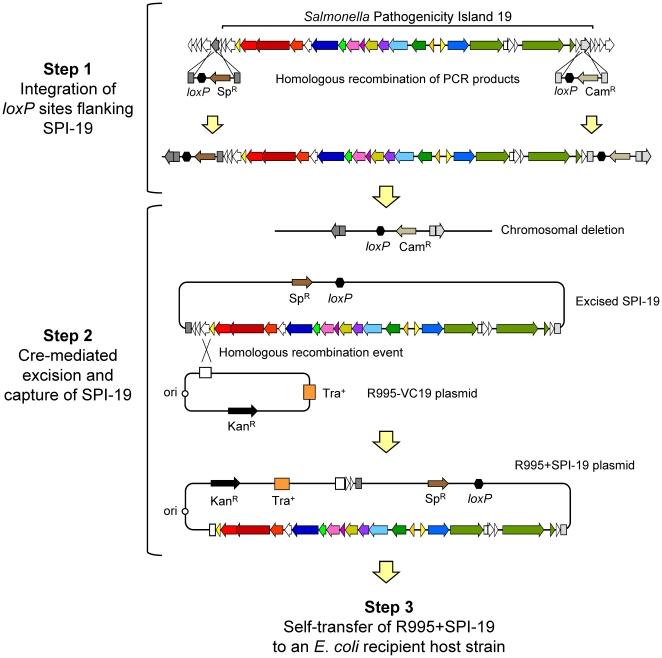
Schematic representation of the capture of SPI-19 from *S.* Gallinarum 287/91 by the VEX-Capture method. Each of the major steps of the procedure is depicted in brackets as detailed in the Materials and Methods section.

The ΔSPI-19 mutant strain was complemented *in trans* with the complete SPI-19 cloned using the Vector-mediated Excision and Capture (VEX-Capture) technique, which allows targeted excision and *in vivo* cloning of large chromosomal regions [25], [26], [27]. Single *loxP* sites were introduced on either side of the SPI-19 in the genome of *S*. Gallinarum strain 287/91 through Lambda-Red recombination [23]. SPI-19 was excised from the bacterial chromosome through transient expression of Cre recombinase, and the excised island was captured through homologous recombination with plasmid R995-VC19, a derivative of the self-transmissible, broad host-range plasmid R995 containing a region of homology to the excised SPI-19. The regions targeted for Cre/*loxP* recombination, as well as the structure of the captured island are shown in **Figure 4** **.** The resulting plasmid (R995+SPI-19) was used to complement our *S*. Gallinarum SPI-19 deletion mutant *in trans* to generate strain ΔSPI-19/R995+SPI-19. This strain was tested in a competitive index experiment against a derivative of the ΔSPI-19 mutant containing an empty copy of the vector, as well as the wild-type parental isolate bearing the empty vector (strains ΔSPI-19/R995 and WT/R995, respectively). White Leghorn chicks were infected orally with an equal mixture of ∼10^9^ CFU of strains WT/R995, ΔSPI-19/R995 and ΔSPI-19/R995+SPI-19 at four days post-hatch. As in previously described experiments, the ileum, ceca, liver and spleen were removed to determine the levels of bacterial colonization at days 1, 3, and 5 post-infection.

**Figure 5:**
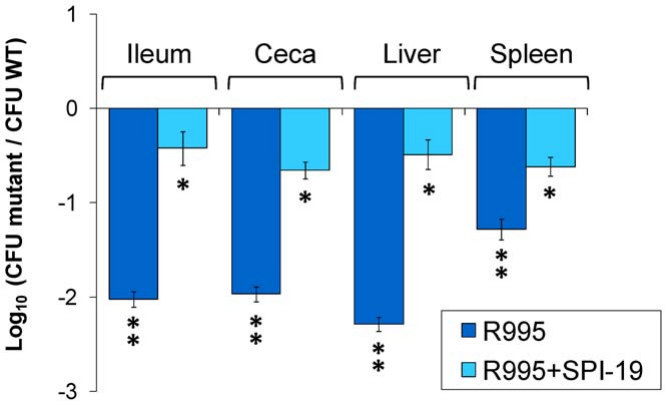
Complementation of the *S*. Gallinarum ΔSPI-19 mutant with SPI-19 *in trans*. Four day old White Leghorn chickens were orally infected with ∼10^9^ CFU of an approximately equal mixture of strains WT/R995, ΔSPI-19/R995 and ΔSPI-19/R995+SPI-19. Data from day 5 post-infection is shown as a representative time-point. Error bars represent standard error. Statistical significance was determined using a two-tailed Student's *t* test, and asterisks indicate that normalized output ratios were significantly statistically different from the equivalent ratio in the inoculum. ** *P* value of<0.001, * *P* value of<0.05.

In our experiments, transfer of the complete SPI-19 region into the ΔSPI-19 mutant strain restored the ability of the mutant isolate to colonize ileum, ceca, liver and spleen directly linking SPI-19 to the colonization defect in our ΔSPI-19 mutant (**Figure 5**). In contrast, the ΔSPI-19 mutant bearing the empty R995 vector was as defective for colonization during competitive infection with the wild-type organism as the ΔSPI-19 mutant itself. These results indicate that SPI-19 contributes to the intestinal and systemic colonization of experimentally infected chicks by *S*. Gallinarum strain 287/91.

### Transfer of SPI-19 from *S.* Gallinarum results in reduced chicken colonization by *S.* Enteritidis

**Figure 6:**
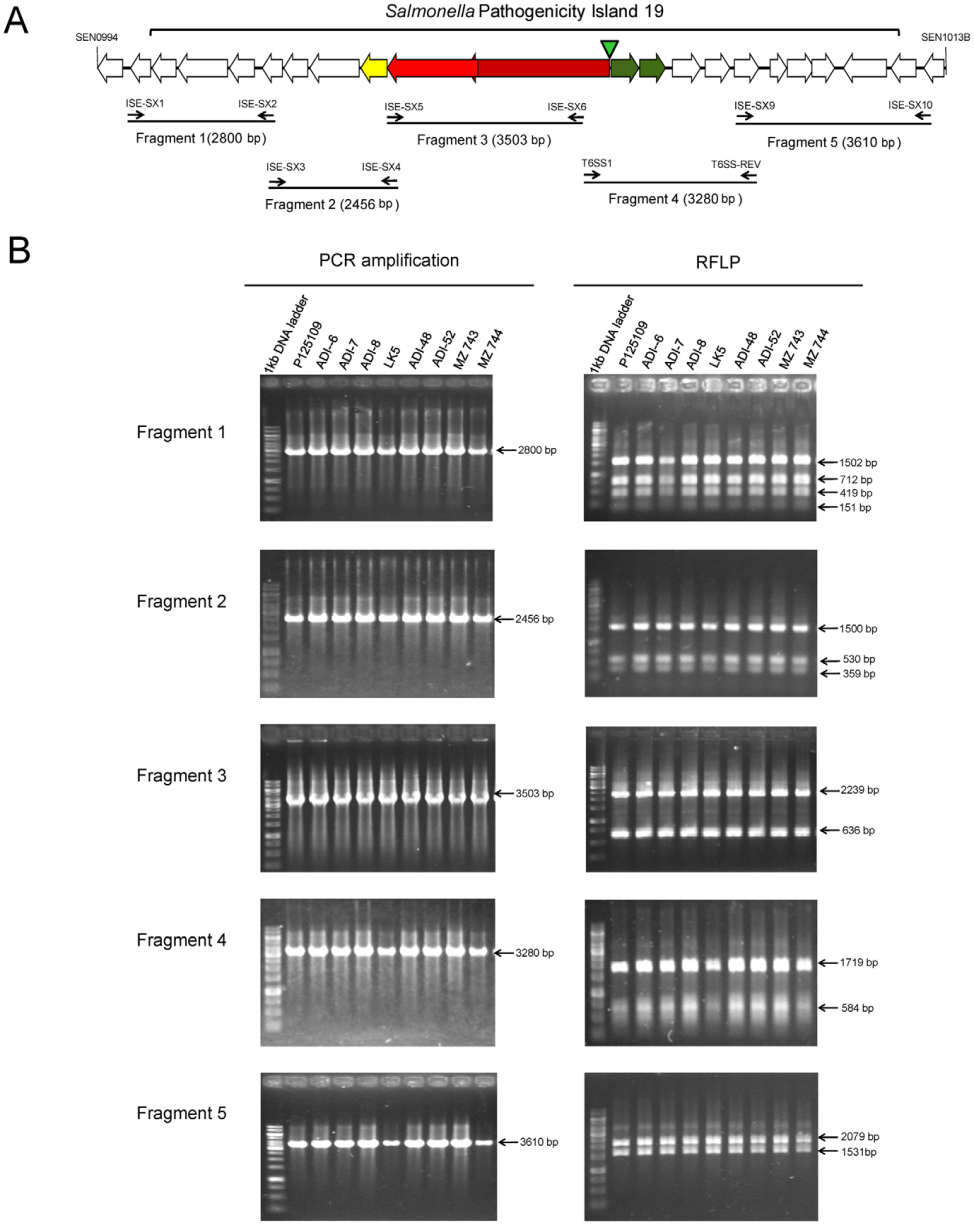
Detection of SPI-19 in the genome of *S*. Enteritidis wild-type isolates. (**A**) Schematic representation of SPI-19 in *S.* Enteritidis. Conserved T6SS components are shown in color as in **Figure 1**. The location of primers designed to amplify 5 fragments of the island by tiling-PCR is shown. A green arrowhead indicates the location of the putative deletion joint-point for SPI-19 in *S*. Enteritidis. (**B**) Tiling-PCR and RFLP analyzes of the 5 fragments of SPI-19 as described in the Materials and Methods section. The figure is representative of the results obtained for a total of 50 *S.* Enteritidis isolates analyzed, including reference strains P125109 and LK5 (**Table S1**).

In the broad host range isolate *S.* Enteritidis P125109 [5], SPI-19 is a degenerate genetic element that has lost a ∼24 kb region that contains most of the genes linked to the T6SS (**Figure 1**) [5], [22]. To determine if this genetic structure is a common feature among *S.* Enteritidis strains, the overall structure of SPI-19 was determined by tiling-PCR and restriction fragment length polymorphism (RFLP) analyses. PCR amplification and RFLP patterns from 50 *S.* Enteritidis isolates studied (of different phage-types, isolation dates and origins) revealed that the overall structure of SPI-19 is conserved, including the internal deletion described for the sequenced PT4 strain P125109 [5], [22]. Representative data from this analysis is shown for a limited number of strains in **Figure 6**. Thus the loss of the T6SS locus encoded in SPI-19 appears to be a common feature of serotype Enteritidis isolates.

**Figure 7:**
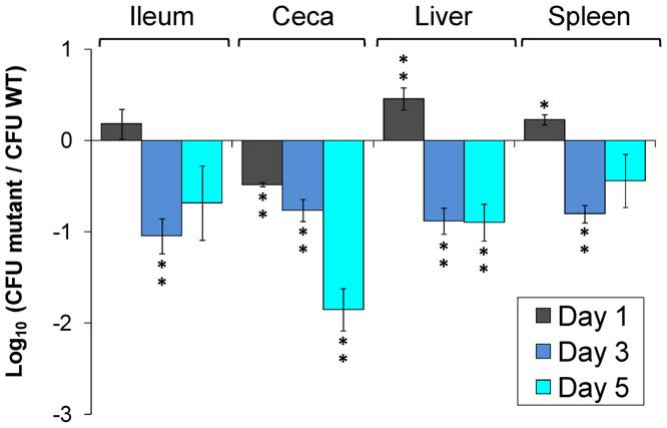
Competitive index experiments with the *S.* Enteritidis R995+SPI-19 strain versus the wild-type strain. Four day old White Leghorn chickens were orally infected with ∼10^9^ CFU of a mixture containing the mutant strains and the wild-type *S*. Enteritidis strain P125109. After 1, 3 and 5 post-infection chicks were humanely euthanized and, ileum, ceca, liver and spleen were recovered, and these tissues were homogenized. Bacterial CFU were determined by serial dilution and plating on appropriate media. Error bars represent standard error. Statistical significance was determined using a two-tailed Student's *t* test, and asterisks indicate that normalized output ratios were significantly statistically different from the equivalent ratio in the inoculum. ** *P* value of<0.001, * *P* value of<0.05.

To assess the impact of carrying a complete T6SS locus on the ability of *S.* Enteritidis to colonize the avian host, plasmid R995+SPI-19 was transferred by conjugation into the *S.* Enteritidis strain P125109 to produce strain P125109/R995+SPI-19. This strain, bearing the complete SPI-19 of *S.* Gallinarum strain 287/91 *in trans,* was tested in a competitive infection assay against the wild-type parental strain containing an empty R995 plasmid (P125109/R995) in our chicken model of infection (**Figure 7**). Surprisingly, the presence of R995+SPI-19 significantly increased the ability of strain P125109 to colonize the ileum, liver and spleen of infected chicks by day 1 post-infection. This colonization advantage was not lasting however, as strain P125109/R995+SPI-19 presented a strong colonization defect for each organ analyzed from day 3 post infection to the conclusion of the experiment. These results suggest that transfer of SPI-19 from *S*. Gallinarum has a negative impact on the ability of *S*. Enteritidis to colonize the avian host.

## Discussion

In this work we studied the contribution of the recently identified SPI-19 to the ability of *S.* Gallinarum to colonize chicks. We demonstrate that SPI-19 contributes to the efficient colonization of the intestinal tract and systemic sites in chicks infected by *S.* Gallinarum strain 287/91. In addition, we provide direct genetic evidence that the T6SS encoded in this island is responsible for this contribution.

Additional experiments revealed that mutations in either SPI-19 or *clpV*, a gene that encodes an essential component for T6SS function, result in a strong colonization defect in the ileum, ceca, liver and spleen of *S*. Gallinarum infected chicks. Complementation of the ΔSPI-19 mutant strain *in trans*, restored the ability of this mutant to colonize the gut and internal organs of infected chickens. We also determined that the R995 plasmid and its derivatives are highly stable *in vivo*, supporting the use of VEX-Capture to study the contribution of *Salmonella* pathogenicity islands *in vivo*.

Lack of SPI-19 or ClpV generated a strong colonization defect for both the gastrointestinal tract and internal organs of experimentally infected chickens. Two possible explanations may account for this global colonization defect. First, SPI-19 might contribute only to the colonization of the gastrointestinal tract of infected chickens. In this scenario, reduced colonization of the ileum and ceca would decrease the bacteria available to breach the epithelial barrier and spread to systemic sites. A second potential explanation is based on experimental evidence indicating that, unlike other *Salmonella* serotypes, *S.* Gallinarum interacts primarily with the gut-associated lymphoid tissue (GALT) rather than with non-phagocytic cells in the chicken gastrointestinal tract [28], [29]. Based on this evidence, any defect in the interaction of *S.* Gallinarum with cells of the GALT or other cell types where the organism may replicate intracellularly, could lead to a reduction in the colonization of both the gastrointestinal tract and the internal organs of infected chickens. Supporting this notion, it has been reported that *S.* Gallinarum SPI-2 mutants defective for intracellular replication and survival inside macrophages *in vitro*, are also defective for colonization of the ileum, ceca, liver and spleen after oral infection of Rhode Island Red chickens [9]. Furthermore, the T6SS encoded in SPI-19 is phylogenetically related to the T6SS encoded in the *vas* gene cluster of *Vibrio cholerae* strain V52 [22], which mediates host-cell cytotoxicity and impairment of phagocytosis by murine macrophages and intestinal inflammation [30], [31].

We demonstrate that lack of either SPI-19 or ClpV results in a strong colonization defect for both the gastrointestinal tract and systemic organs (just as described for a *S.* Gallinarum SPI-2 mutant). It is therefore plausible that the T6SS encoded in SPI-19 is potentially important for the interaction of *S.* Gallinarum with cells of the GALT and/or for interaction with those cells where Gallinarum may establish a niche for growth during infection. Future studies will focus on the mechanisms of such possible interactions.

We did not observe the appearance of clinical manifestations of fowl typhoid by the end of our experimental time points in our chick model after infection with the sequenced *S*. Gallinarum isolate 287/91. Although we show that SPI-19 contributes to *S.* Gallinarum colonization of the gut and internal organs of infected chickens, we could not determine the contribution of SPI-19 to the development and severity of the fowl typhoid disease *per se*. This could have been because the chosen experimental end point (5 days post infection) was not long enough for the disease to develop or because the 287/91 sequenced strain may have lost some of its virulence potential. Further work will be needed to establish if the contribution of SPI-19, and the T6SS encoded therein, to the colonization of the chicken corresponds to a widespread phenomenon among other *S*. Gallinarum virulent isolates and if SPI-19 contributes to the development of fowl typhoid.

In this work we also determined that the loss of the T6SS encoded in SPI-19 in *S.* Enteritidis is not restricted to the sequenced strain, as we detected the same overall genetic structure for SPI-19 in 50 *S.* Enteritidis isolates irrespective of their phage-type, date and isolation origin. These findings suggest that loss of the SPI-19 T6SS is a conserved feature of this serotype. Genome-wide analyses indicate that *S.* Gallinarum is a direct descendant of *S.* Enteritidis [5]. In this scenario, the presence of a complete SPI-19 in S. Gallinarum suggests that degeneration of the island in *S.* Enteritidis must have occurred after the divergence of both serotypes from their common ancestor.

Transfer of a complete SPI-19 from *S.* Gallinarum into *S.* Enteritidis increased the initial ability of the bacterium to colonize the ileum, liver and spleen of infected chickens but a strong colonization defect was observed after three days post infection. We envision two possible explanations for this observation. First, it is possible that the intact T6SS could be secreting and/or translocating substrates in an unregulated manner in *S.* Enteritidis and this deregulation would have a negative impact on the overall fitness of the bacterium. This might explain the ability of the *S*. Enteritidis isolate bearing the intact T6SS to colonize well at the early time point, but improperly regulated expression of the T6SS at later stages might result in the strong colonization defects we observed. A similar hypothesis has been proposed to explain the intracellular survival defect in macrophages observed for a *S*. Typhimurium ΔSPI-2 mutant carrying the complete SPI-2 cloned in plasmid R995 by VEX-Capture [25].

Alternatively, transfer of a functional T6SS to *S.* Enteritidis may have a negative impact on colonization *per se* and that loss of this system has conferred to this serotype an advantage towards the avian host. In contrast to *S.* Gallinarum, colonization of the gastrointestinal tract by *S.* Enteritids generates an inflammatory reaction characterized by production of pro-inflammatory chemokines and cytokines followed by infiltration of heterophils and macrophages [32], [33]. If the SPI-19 T6SS is indeed involved in the interaction of *Salmonella* with phagocytic cells, as suggested above, its transfer into *S.* Enteritidis may result in increased cytotoxicity and intracellular bacterial survival capabilities. This increase could have a negative impact for the bacterium as it could result in a stronger host immune response leading to a faster clearance. *S.* Gallinarum would not have the same problem as it has been proposed to have developed an initial “stealth strategy” mainly due to the absence of flagella [32]. We are currently evaluating these hypotheses to determine the mechanisms responsible for the phenotypes observed.

In conclusion, we have shown that SPI-19 contributes to the efficient colonization of experimentally-infected chickens by *S*. Gallinarum and provide genetic evidence that the T6SS encoded therein is responsible for these phenotypes. In addition, we show that the presence of a complete T6SS locus in *S.* Enteritidis has an overall negative impact in the ability of the bacterium to colonize efficiently the chicken. The functionality of the T6SS encoded in SPI-19, as well as the molecular mechanisms by which it contributes to *Salmonella* pathogenesis and host-specificity, are currently under study in our laboratory.

## Materials and Methods

### Ethics Statement

All procedures described in this work were approved by the Texas A&M University Institutional Animal Care and Use Committee (TAMU AUP# 2007-18) and were performed in accordance with the Guide to the Care and Use of Laboratory Animals, the Public Health Service Policy on the Humane Care and Use of Laboratory Animals.

### Bacteria and growth conditions

The bacterial strains used in the present study are listed in **Table 1**. Information on 50 wild-type *S.* Enteritidis isolates analyzed can be found in **Table S1**. Bacteria were routinely grown in Luria-Bertani (LB) medium (10 g/l tryptone, 5 g/l 128 yeast extract, 5 g/l NaCl) at 37°C with aeration. LB medium was supplemented with ampicillin (Amp; 100 mg/l), chloramphenicol (Cam; 20 mg/l), kanamycin (Kan; 50 mg/l), trimethoprim (Tmp; 100 mg/l) and spectinomycin (Sp; 250 mg/l) as appropriate. Media were solidified by the addition of agar (15 g/l).

**Table 1:** Bacterial strains used in this study.

Strain	Features	Source or reference
***Escherichia coli***		
DH5α	F^−^ Φ80*lacZ*ΔM15 Δ (*lacZYA-argF*)U169 *deoR recA1 endA1 hsdR17*(r_k_^−^, m_k_^+^) *phoA supE44 thi-1 gyrA96 relA1* λ^−^	Laboratory collection
EC100D *pir*-*116*	F^−^ *mcrA* Δ (*mrr-hsdRMS-mcrBC*) Φ*80lacZ*ΔM15 Δ*lacX74 recA1 endA1 araD139* Δ (*ara, leu*)*7697 galU galK λ- rpsL* (Str^R^) *nupG pir-116 (dhfr)*	Laboratory collection
EC100D *pir*-*116*/R995+SPI-19	EC100D *pir*-*116* + R995::SPI-19 (*SG1021*-*SG1056*)	This work
DH5α/R995	DH5α + R995 Vex Capture Vector	This work
DH5α/R995-VC19	DH5α + R995::*SG1022*	This work
***Salmonella*** ** Gallinarum**		
287/91	Wild-type, sequenced isolate (NCTC13346)	Laboratory collection [5]
287/91 ΔSPI-19	287/91 ΔSPI-19 (*SG1024*-*SG1054*)	This work
287/91 Δ*clpV*	287/91 Δ*SG1034*	This work
287/91/R995	287/91+ R995 Vex Capture Vector	This work
287/91 ΔSPI-19/R995+SPI-19	287/91 ΔSPI-19 (*SG1024*-*SG1054*) + R995::SPI19 (*SG1021*-*SG1056*)	This work
***Salmonella*** ** Enteritidis**		
P125109	Wild-type, sequenced isolate (NCTC13349)	Laboratory collection [5]
P125109/R995	P125109 + R995 Vex Capture Vector	This work
P125109/R995+SPI-19	P125109 + R995::SPI19 (*SG1021*-*SG1056*)	This work

### Standard DNA techniques

Total genomic DNA was obtained from overnight bacterial cultures using the “GenElute Bacterial Genomic DNA” kit (Sigma) according to the manufacturer's instructions. Plasmid DNA was obtained from overnight cultures using either the “QIAprep Spin Miniprep Kit” or the “QIAprep Spin plasmid Maxi Kit” (QIAGEN), according to the manufacturer's instructions. PCR products were purified using the “QIAquick PCR Purification Kit” (QIAGEN) as recommended. Restriction digests using endonucleases *Sau*96I, *Ssp*I, *Bam*HI, *Eco*RI and *Xba*I (Fermentas) and ligations using the T4 DNA ligase (NEB) were conducted as recommended by the manufacturers. DNA samples were routinely analyzed by electrophoresis in 1% agarose gels (0.5× TAE buffer) and visualized under UV light after ethidium bromide staining.

### PCR amplifications and RFLP analyses

Primers for PCR amplification were designed using the “Vector NTI Advance 11.0” software. PCR amplifications were performed using a “MultiGene TC9600-G” thermal cycler (Labnet) and *GoTaq Flexi* DNA polymerase (Promega). Reaction mixes contained 1×buffer, 2 mM MgCl_2_, 200 µM of each deoxynucleoside triphosphate, 200 nM of each primer, 50–100 ng of template DNA, and 0.5 U of DNA polymerase. Standard conditions for amplification were: 3 min at 94°C, followed by 30 cycles of incubations at 94°C for 30 s, 55°C for 30 s, and 72°C for 2 min, followed by a final extension step at 72°C for 5 min.

For tiling-PCR analyses, a set of 10 primers were designed based on the reported sequence of *S.* Enteritidis strain P125109 to amplify 5 fragments of SPI-19 presenting overlapping sequences of ∼50–200 bp (**Figure 6** and **Table 2**). Conditions for tiling-PCR amplification were: 3 min at 94°C, followed by 30 cycles of incubations at 94°C for 30 s, 58°C for 30 s, and 72°C for 4 min, followed by a final extension step at 72°C for 7 min. The amplification products were analyzed by electrophoresis in 1% agarose gels.

**Table 2:** Oligonucleotide primers used in this study.

Primer	Sequence a
**Tiling-PCR**	
ISE-SX1	CTTTGTTCCCATCCTGATGT
ISE-SX2	AGTTATAATGGCGGAGGAGA
ISE-SX3	TTTCCTGTTGCAGAGGTAAA
ISE-SX4	AATTAATGAACGGCTGGATG
ISE-SX5	TTCAGCAACAGCCAGCGGCT
ISE-SX6	GCCGGTTGTCCGTGTTTTCC
T6SS1	CAGGTTGCCATAATCGTCCA
T6SS-REV	TTGTGAAGGCGTTCAAGTTC
ISE-SX9	ATCTCAATGGAGATGACACA
ISE-SX10	ACTGCACATAGCAAGCTTTA
**Mutagenesis**	
SG1034_(H1+P1)	ATGATCCAGATTGACTTAGCCACCCTGGTAAAACGGCTTG*GTGTAGGCTGGAGCTGCTTC*
SG1034_(H2+P2)	TCATAGAACGGCTTCGTCCTCTGCTTCCGCTACGGGCTGA*CATATGAATATCCTCCTTAG*
SG1034_OUT5	ATCCGGCATGTTCTTGCG
SPI-19_(H1+P1)	TAGCTGAATTGCAATATGCGAAAAAAGCCGAGCTTGATGACAAAC*GTGTAGGCTGGAGCTGCTTC*
SPI-19_(H2+P2)	AAGCATCTTCAATAATCACGGGTATAAATGCTTACACTCTTTATC*CATATGAATATCCTCCTTAG*
C3	CAGCTGAACGGTCTGGTTATAGG
**VEX Capture**	
SG1021_VEX_H1_U1	GGGTTTTATTGTGATAGCAAAATTCTTCCCGTGGTATAGC*GGCCACGTGGGCCGTGCACCTTAAGCTT*
SG1021_VEX_H2_U2	TTATCCTCTATTAAAATTCTTTTGTTGATTGAAAACTTTC*CAGGTCGACGTCCCATGGCCATTCGAATTC*
SG1056_VEX_H1_D1	AGCGGAGCGACAGCTCCTCTGGATTTATGTCAGCGGAGA*GGTTTAACGGTTGTGGACAACAAGCCAGGG*
SG1056_VEX_H2_D2	CTACCAGTAAACGAACAGTGCCAAATACGGAGCCGGTGGA*CAGGTCGACGTCCCATGGCCATTCGAATTC*
SG1022_VC_OUT5	GCTCTAGAATGATGACGAAATACGGTGT
SG1022_VC_OUT3	GCTCTAGATAGCCAAGAATTTCAGTGAG
5trfA	ACGTCCTTGTTGACGTGGAAAATGACCTTG
3trfA	CCGGAAGGCATACAGGCAAGAACTGATCG
SPI19_OUT_UP	CGCCTTACATAGCTACGATCTCAGG
SPI19_OUT_DOWN	CAGCTCAGGCAAAGAACCTATGC

^*a*^Italics indicate the region that anneals to the 5′ or 3′ end of the antibiotic resistance cassette used for the mutagenesis. Underline indicates *Xba*I restriction sites used for cloning in plasmid R995.

PCR products from the 5 fragments were further analyzed by RFLP using specific restriction endonucleases. Fragment 1 (2800 bp) was digested with *Sau*96I which generates predicted products of 16, 151, 419, 712, 1502 bp. Fragment 2 (2456 bp) was digested with *Ssp*I which generates predicted products of 56, 359, 530, 1511 bp. Fragment 3 (3503 bp) was digested with *Bam*HI which generates predicted products of 628, 636, 2239 bp. Fragment 4 (3280 bp) was digested with *Sau*96I which generates predicted products of 186, 336, 455, 584 y 1719 bp. Fragment 5 (3610 bp) was digested with *Eco*RI which generates predicted products of 1531 and 2079 bp. Each digestion was analyzed by electrophoresis in 1% agarose gels (0.5× TAE buffer) and visualized under UV light after ethidium bromide staining. Tiling-PCR and RFLP analyses were performed for each of the 50 *S*. Enteritidis isolates described in **Table S1**.

### Mutant constructions and analyses

Mutant strains with specific deletions of SPI-19 (*SG1024* to *SG1054*) or *clpV* (*SG1034*) gene and the concomitant insertion of a Cam-resistance cassette were constructed using the Lambda Red recombination method [23]. PCR primers 60 bases long were synthesized with 40 nt on the 5′ ends corresponding to the ends of the desired deletion (**Table 2**) and the 3′ 20 nt of each primer was annealed to the 5′ or 3′ end of a Cam resistance cassette flanked by the FRT sites (Flp recombinase target sequence) present in plasmid pCLF2 (GenBank accession number HM047089). PCR amplifications were carried out under standard conditions using pCLF2 as template DNA.

*S.* Gallinarum strain 287/91 carrying the temperature-sensitive plasmid pKD46, which expresses the Lambda-Red recombinase system, was grown to an OD_600_ of 0.5 at 30°C in LB medium containing Amp and L-arabinose (10 mM). Bacteria were made electrocompetent by sequential washes with ice-cold sterile 10% glycerol, and transformed with approximately 500 ng of each purified PCR product. Transformants were selected on LB agar plates containing Cam at 37°C. The presence of each mutation was confirmed by PCR amplification using primers flanking the sites of substitution. Each mutant was finally assayed for Amp sensitivity to confirm the loss of pKD46.

To obtain non-polar deletions, the Cam-resistance gene was removed by transforming each mutant with pCP20, which encodes the FLP recombinase. Transformants were plated on LB agar at 37°C. Individual colonies were replica-plated on LB agar, LB agar containing Amp and LB agar containing Cam. The plates were incubated at 42°C. Transformants that had lost the resistance cassette and plasmid pCP20 were selected as those colonies that were able to grow only on LB agar. The absence of the antibiotic-resistance gene cassette was confirmed for each mutant by PCR analysis.

### Cloning and transfer of *S.* Gallinarum strain 287/91 SPI-19 region

Cloning of SPI-19 from the *S.* Gallinarum 287/91 genome onto plasmid R995 was performed using a modification of the VEX-Capture method [26]. The procedure is schematized in **Figure 4** and consists of three major steps. Step 1: Single *loxP* sites were inserted flanking the targeted ∼42 kb genomic region by recombination of PCR products using a modification of the Lambda-Red recombination method [23]. PCR primers 60 bases long were synthesized with 40 nt on the 5′ ends corresponding to the ends of ORFs SG1021 or SG1056 (**Table 2**) and the 3′ 20 nt of each primer was annealed to the 5′ or 3′ end of an antibiotic resistance cassette including a *loxP* site (Cre recombinase target sequence) present in plasmids pVEX1212 (Sp resistance cassette) and pVEX2212 (Cam resistance cassette). Insertion of *loxP* sites in the chromosome was followed by selection using the appropriate antibiotic and confirmed by PCR analyses using primers SPI19_OUT_UP and SG1021_VEX_H2_U2 for the upstream *loxP* insertion, and primers SPI19_OUT_DOWN and SG1056_VEX_H2_D2 for the downstream *loxP* insertion (**Table 2**). Step 2: A plasmid harboring an internal region of SPI-19 was constructed in order to capture the excised SPI-19 by a homologous recombination event. PCR primers SG1022_VC_OUT5 and SG1022_VC_OUT3 were designed to amplify a 1,106 bp region internal to ORF SG1022 flanked by *Xba*I restriction sites. The PCR product was cloned into the unique *Xba*I site in R995 to generate plasmid R995-VC19. Correct cloning of *SG1022* into R995 was confirmed by PCR analyses. *E. coli* strain DH5α was transformed with both plasmids R995-VC19 and pEKA30, a mobilizable broad-hostrange IncQ plasmid which constitutively expresses the Cre recombinase. Both plasmids were transferred by conjugation to the derivative of *S*. Gallinarum strain 287/91 carrying SPI-19 flanked by *loxP* sites. In the presence of plasmid pEKA30, the SPI-19 was excised from the chromosome as a non-replicating, circular molecule via Cre-mediated site specific recombination. The circular intermediate was then captured by homologous recombination with plasmid R995-VC19, generating plasmid R995+SPI-19. Step 3: Plasmid R995+SPI-19 was transferred from *S.* Gallinarum to the *E. coli* recipient strain EC100D *pir*-*116* by conjugation. The resulting strain was used as donor for subsequent transfer of plasmid R995+SPI-19 to *S.* Enteritidis strain P125109 and derivatives of *S.* Gallinarum strain 287/91 by conjugation. Plasmid DNA was isolated from each recipient strain carrying plasmid R995+SPI-19 and the presence of the SPI-19 region was confirmed by visualization of supercoiled plasmid DNA in agarose gel, and by PCR analyses with primers used for tiling-PCR of SPI-19 and primers 5trfA and 3trfA, which amplify an internal region of plasmid R995 (**Table 2**).

For competitive infections in chickens, the *in vivo* stability of plasmids R995 and R995+SPI-19 was assessed in each organ at each time point studied. Since each strain tested harbored different chromosomal antibiotic resistance cassettes, different antibiotic combinations were used as indicators if these plasmids were being lost from the bacterial population. No differences were observed in the bacterial counts from different antibiotic combinations tested, indicating that R995 and its derivatives are highly stable *in vivo*. In addition, presence of an empty copy of plasmid R995 had no impact in the ability of *S.* Gallinarum to efficiently colonize the chickens, as bacterial counts from the gut and internal organs of chickens infected with WT/R995 were equivalent to the wild-type *S.* Gallinarum strain 287/91.

### Chick Inoculation

SPF White Leghorn eggs were obtained from Charles River SPAFAS. Eggs were incubated for 21 days in a Sportsman Incubator as per the manufacturer's instructions. After hatching, chicks were housed in a poultry brooder in groups of four with *ad libitum* access to tap water and irradiated lab chick diet. Brooder temperature was maintained at 32°C to 35°C.

All infections were carried out essentially as previously described [34]. For single infection, groups of 12 White Leghorn chicks were inoculated orally with ∼10^9^ CFU of wild-type *S.* Gallinarum strain 287/91 at four days post hatching. Uninfected control birds were housed separately from infected birds to minimize cross contamination. Four birds from the infected group and 2 from the control group were euthanized by CO_2_ asphyxiation on days 1, 3 and 5 post-infection. Liver, spleen, sections of ileum and both cecal arms (cecum plus contents) were collected. A portion of each collected organ was weighed and homogenized in 2 ml sterile PBS. Following homogenization, samples were serially diluted and plated on LB agar plates containing the appropriate antibiotics for determination of CFU.

For competitive infection, groups of 12 White Leghorn chicks were inoculated orally with ∼10^9^ CFU of an equal mixture of the strains to be tested in a volume of 0.1 ml of PBS. The inoculum was serially diluted and plated on LB agar plates containing the appropriate antibiotics for determination of CFU and the exact input ratio. Four birds from the infected group and 2 from the control group were euthanized by CO_2_ asphyxiation on days 1, 3 and 5 post-infection. Liver, spleen, sections of ileum and both cecal arms (cecum plus contents) were collected. A portion of each collected organ was weighed and homogenized in 2 ml sterile PBS. Following homogenization, samples were serially diluted and plated on LB agar plates containing the appropriate antibiotics for determination of CFU.

### Statistical analysis

Data obtained from competitive infection experiments were calculated as a mean ratio of mutant to wild-type, normalized to the input ratio and converted logarithmically. Error bars denote standard error. Statistical significance was determined using a two-tailed Student's *t* test. *P* values of<0.05 were considered statistically significant (SPSS software, SPSS, Inc., Chicago, IL).

## Supporting Information

Table S1 List of wild-type *S*. Enteritidis isolates used in this study. This table includes information on 50 wild-type *S*. Enteritidis isolates analyzed in this study.
(0.04 MB XLS)
